# The application of unsupervised deep learning in predictive models using electronic health records

**DOI:** 10.1186/s12874-020-00923-1

**Published:** 2020-02-26

**Authors:** Lei Wang, Liping Tong, Darcy Davis, Tim Arnold, Tina Esposito

**Affiliations:** 1 School of Statistics, Renmin University of China, 59 Zhong Guan Cun Ave, Hai Dian District, Beijing, People’s Republic of China; 2 Department of Mathematics, Statistics, and Computer Science, University of Illinois at Chicago, 851 S Morgan St, Chicago, IL 60607 USA; 3 Advocate Aurora Health, 3075 Highland Parkway, Downers Grove, IL 60515 USA; 4 Cerner Corporation, 2800 Rockcreek Parkway, North Kansas City, MO 64117 USA

**Keywords:** Autoencoder, LASSO, Enhanced Reg, Predictive model, Predictive performance, Important response-specific predictors

## Abstract

**Background:**

The main goal of this study is to explore the use of features representing patient-level electronic health record (EHR) data, generated by the unsupervised deep learning algorithm autoencoder, in predictive modeling. Since autoencoder features are unsupervised, this paper focuses on their general lower-dimensional representation of EHR information in a wide variety of predictive tasks.

**Methods:**

We compare the model with autoencoder features to traditional models: logistic model with least absolute shrinkage and selection operator (LASSO) and Random Forest algorithm. In addition, we include a predictive model using a small subset of response-specific variables (Simple Reg) and a model combining these variables with features from autoencoder (Enhanced Reg). We performed the study first on simulated data that mimics real world EHR data and then on actual EHR data from eight Advocate hospitals.

**Results:**

On simulated data with incorrect categories and missing data, the precision for autoencoder is 24.16% when fixing recall at 0.7, which is higher than Random Forest (23.61%) and lower than LASSO (25.32%). The precision is 20.92% in Simple Reg and improves to 24.89% in Enhanced Reg. When using real EHR data to predict the 30-day readmission rate, the precision of autoencoder is 19.04%, which again is higher than Random Forest (18.48%) and lower than LASSO (19.70%). The precisions for Simple Reg and Enhanced Reg are 18.70 and 19.69% respectively. That is, Enhanced Reg can have competitive prediction performance compared to LASSO. In addition, results show that Enhanced Reg usually relies on fewer features under the setting of simulations of this paper.

**Conclusions:**

We conclude that autoencoder can create useful features representing the entire space of EHR data and which are applicable to a wide array of predictive tasks. Together with important response-specific predictors, we can derive efficient and robust predictive models with less labor in data extraction and model training.

## Background

In recent years, there has been increasing interest in clinical prediction research as well as a growing consensus on the importance of predictive models for medical science [1–5]. Predictive models can be used to aid in the clinical decision-making process, inform the potential development of illness, or relate the current health status of individuals to their future possible outcomes. The dramatic increase of EHR (Electronic Health Record) data provides many novel opportunities to capture the association between patient outcomes and clinical treatments, while also pushing the dimensionality and complexity of data to a state where some classical predictive models may fail. LASSO (Least Absolute Shrinkage and Selection Operator) [6], one of the most popular variable selection methods, has been a good solution to prediction problems for high dimensional data by shrinking small coefficients to zero during regression. In practice, when the response variable has a binary outcome, logistic models are typically applied with LASSO variable selection. Machine learning procedures such as Random Forest [7] have also been successfully implemented in various practical problems. Operating on the divide and conquer principle, Random Forest exhibits remarkably good results by averaging the results obtained from a predefined number of randomized individual decision trees while requiring very little tuning [8].

LASSO and Random Forest are both supervised strategies that usually use different sets of predictors for models with different response variables. Even for the same response, such as a readmission event, the predictors can vary widely across studies. Miotto et al. [9] proposed a data representation using an unsupervised deep learning method – a three-layer stack of denoising autoencoders – which has the potential to generate standardized features to represent the original EHR data and can be efficiently used in various types of predictive models. The innovative work by Miotto et al. inspired us to probe into some possible applications of autoencoder in predictive models using EHR data.

Autoencoder has been successfully used in word sequence processing [10], human pose image recovery [11], and nuclei detection of breast cancer histopathology images [12], among many other applications. It is a process exerting nonlinear transformations layer by layer during which the linear combinations of output from the former layer become the input of nonlinear activation functions in the following layer. The objective of autoencoder is to minimize the difference between final outputs and inputs from the first layer while prioritizing the most useful information instead of simply compressing or copying raw data [13]. That implies the usage of this strategy is to capture the most informative data while reducing noise. If the dimension of the last hidden layer is less than the number of original predictors, a lower dimensional data representation is obtained which can function as a new feature set in predictive models, consequently mitigating the downsides of high dimensionality. Therefore, in this paper, we research a strategy that deploys the lower-dimensional data representation to reduce the feature set size when building classifiers for EHR data. It is worth noting that, due to its unsupervised nature, the lower dimensional representation is capable of being applied to various models with different response variables. Though it requires massive computation, the process of extracting new features can be computationally efficient with the machine learning platform H2O which takes advantage of distributed systems and in-memory computing [14].

In this paper, we first use simulated data to explore the applicability of new features from autoencoder to predictive models under different handlings of data for quantitative variables and missing values. The application of EHR data raises questions about the validity and impact of some conventional practices when processing clinical data, such as categorizing numeric variables and the handling of missing values. Categorization may cause serious information loss and improper critical values may lead to additional bias [15–17]. However, categorization also simplifies the data and eventually the predictive model. Missing data is a common problem in real world data and is widespread in EHR data [3, 18–21]. There are many options to address missing data with less loss, including simple, widely used strategies like single imputation or coding missing data as unknown. In literature, there has been a lot of research on the effects of categorization and missing data through either simulation or real data analysis [22–26]. Here we do not focus on quantifying the two effects. Instead, we investigate their influence on various predictive strategies because robustness to data limitations is crucial for EHR applicability. Later, we also use EHR data to build models to predict 30-day readmission rates (Readmit30) and the presence of diseases such as Acute Myocardial Infarction (AMI), Heart Failure (HF), Chronic Obstructive Pulmonary Disease (COPD) and Pneumonia from the patient’s latest clinical visit. More specifically, we fit logistic regression with new features from autoencoder both with and without response-specific important variables as predictors for both simulated data and real data. For better illustration, LASSO, Random Forest, logistic models with only important variables (Simple Reg) and with both important variables and features from autoencoder (Enhanced Reg) are included in the comparison.

## Methods

Simulation study is shown to be a useful tool in the sense that it can be tailored to test the properties of the methods under circumstances which may not be reflected or available in existing real data sets. To investigate the proposed strategy thoroughly, we perform the study first on simulated data that mimics real world EHR data and then on actual EHR data.

### Set up of simulated data

The sample size is set to be 100,000. Assume that the simulated data consists of 100 predictors (*x*_1_, …, *x*_100_), including 72 numeric predictors with multistage influence and 28 numeric predictors with continuous influence. Here continuous influence refers to the predictor that affects response with a single continuous function and multistage influence refers to the predictor that affects response with a piece-wise continuous function which allows a predictor to affect response differently at different stages. We will explain in more detail in the next paragraph. Among all, 12 predictors are assumed to have a significant contribution to the response, including 8 multistage predictors and 4 continuous predictors. The remaining 88 are minor predictors with comparatively smaller contributions to the response. Note that the numbers 100, 72, 28, 12, 8, 4 and 88 are assumed for convenience according to our experience in EHR data and can be changed to some other number without affecting much of the major results of the study. The probability of binary response variable Y being 1 is derived from a logistic model $P\left(Y=1|{x}_1,\dots, {x}_{100}\right)=\exp \left({\sum}_{j=1}^{100}f\left({x}_j\right)\right)/\left(1+\exp \left({\sum}_{j=1}^{100}f\left({x}_j\right)\right)\right)$, where *f*(*x*_*j*_) is the contribution of the j^th^ predictor *x*_*j*_ to the response. It is a flexible and reasonable way to assume the contribution of each predictor is additive [27].

In our model, predictors contribute to response in two ways: multistage and continuous. The consideration of multistage is based on the observation that some quantitative EHR features might exhibit non-linear influence. That is, the two abnormal levels, above or below normal range, can correlate with different health concerns and both might result in hospitalization. For instance, the normal level of serum calcium usually ranges from 8.5 to 10.5 mg/dl if ignoring measurement errors across instruments [28]. Hypocalcemia, the state of low-level serum calcium, often leads to tetany, convulsive seizures, and cardiovascular, psychiatric, and a variety of ectodermal effects. Conversely, hypercalcemia, the state of high-level calcium in blood, is usually related to soft tissue calcification, tubulointerstitial nephropathy, anorexia, nausea, electrocardiographic disturbances, and a spectrum of neurologic changes from headache to coma [28]. This phenomenon inspired us to use a piecewise multistage function to distinguish the possibly distinct intervals. However, there may be a few predictors like age for which we do not witness some clear change points of corresponding influence. Thus, we use a continuous function to approximate the effect of these predictors. A detailed description of the multistage functions (Figure 1) and continuous functions (Figure 2) we have used can be found in the appendix.

All predictors are generated from a multivariate normal distribution, where the mean vector is drawn from a uniform distribution ranging from 150 to 250 with the covariance matrix being *σ*_*i*_*σ*_*j*_0.5^|*i* − *j*|^, where *σ*_*i*_ and *σ*_*j*_ are standard deviations of predictor *x*_*i*_ and *x*_*j*_ respectively, with *σ*_*i*_ and *σ*_*j*_ generated from a uniform distribution *U*[70, 100]. For example, correlation between *x*_1_ and *x*_2_ is 0.5^|1 − 2|^ = 0.5 and between *x*_1_ and *x*_3_ is 0.5^|1 − 3|^ = 0.25. This correlation structure has the convention that more correlated predictors are likely to be put in adjacent columns of the data matrix.

We randomly chose 70% of observations as training data and the rest as testing data. The whole process was repeated 100 times. The mean of AUC (area under the receiver operating characteristic curve) of the true model is 0.7963. The mean of AUC of the true model containing only the 12 important predictors is 0.7353. The mean of AUC of the true model containing only the remaining 88 minor predictors is 0.6899. There are on average 13,265 positive responses out of 100,000 observations, which is designed to mimic the 30-day readmission rate in real data.

### Simulation study design

Here we consider 4 scenarios to handle the simulated data.

1. Raw data: derive models using raw predictors.
2. Correct categories: all numeric predictors are recoded to be categorical with correct critical values. The correct threshold(s) for predictor *x*_*j*_ are the value(s) at which *f*(*x*_*j*_) is equal to zero.
3. Incorrect categories: all numeric predictors are categorized but 12 important predictors and half of minor predictors are recoded according to incorrect cutoff points. Since we usually have certain knowledge about the nature of explanatory variables, the shift of cutoff points from the truth should not be too much. More specifically, the maximum deviation of incorrect thresholds from true critical values is 80, which is assumed to mimic mild but not extreme misclassification mistakes in practice.
4. Incorrect categories and missing data: all important predictors and 90% of the trivial predictors have values missing-at-random conditional on category; the missing percentages for predictors in and out of normal range are 90 and 5% respectively. This missing pattern of normal and abnormal predictor values is intended to approximate real world data such as lab values. All missing observations are set to be an unknown category. In addition, important predictors and 50% of the minor predictors have mis-specified threshold(s) consistent with scenario 3 for observed values.

Scenario 2 is designed to investigate the impact of categorization on predictive models when all predictors are classified correctly. Scenario 3 provides a more realistic version of handling data, as in many situations it is not clear what are the best thresholds for categorization. Scenario 4 is closest to the reality of EHR data, considering the impact of both missing data and categorization. As mentioned in [21], there exist more complex imputation methods like multiple imputation or model-based imputation. However, we consider only the naive method to test robustness of predictive models in extreme cases.

### Real data preparation

We extracted the EHR data from eight Advocate Aurora Health hospitals located in the Chicago metropolitan area. The original data set has 104,398 observation rows with 496 predictors. Redundant variables that are irrelevant or represent extremely rare events were removed. After recoding categorical predictors and lab measurement values to dummy variables, we ended up with 469 predictors. The thresholds for categorization are all based on expert knowledge. Missing values in any predictor were classified as an additional unknown category. Out of the 469 predictors, 8 are numeric, including age, length of stay in the hospital, etc. The rest are all binary variables. Due to strong correlation among predictors like repeated measures for the same person at different time points, the rank of the design matrix is 420, less than the number of predictors. Five response variables are considered in the data: Readmit30, COPD, AMI, HF and Pneumonia. Out of the 104,398 encounters, the number of events for Readmit30 is 12,211 (11.70%), for COPD 7367 (7.06%), for AMI 2288 (2.19%), for HF 6362 (6.09%) and for Pneumonia 3482 (3.34%).

We randomly chose 70% of observations to be the training set and the remainder to be the testing set in 100 repetitions. The list of important response-specific predictors for Readmit30 was derived from prior readmission research [29, 30]. A description of these variables is given in Table 1. The lists of important variables for the other response variables were extracted from 30 training sets using stepwise selection in SAS 9.4. The inclusion criteria for the list of important variables is the highest frequency of being selected as well as a *p* value less than 0.0001 in the final model. In practice, important response-specific variables can also be obtained from literature, expert suggestions, or both.

**Table 1:** Descriptive statistics of important variables for Readmit30. For binary variables like Acuity, the figures represent the number of positive cases and corresponding percentage of the sample (in parenthesis). For numeric variables like Length of Stay, the figures are sample means and corresponding standard deviations (in parenthesis)

Variables	Overall Index Admissions *n* = 104,398	Index Admissions by the Value of Readmit30	
		YES (11.70%) *n* = 12,211	NO (88.30%) *n* = 92,187
1. Length of Stay	4.45 (4.45)	5.61 (5.47)	4.30 (4.27)
2. Acuity	81,048 (77.63)	10,641 (87.14)	70,407 (76.37)
3. Number of ER Encounters in Last Six Months	0.36 (0.91)	0.58 (1.28)	0.33 (0.85)
4. Number of Inpatient Encounters in Last Year	6.01 (12.84)	12.04 (19.83)	5.21 (11.36)
5. Polypharmacy	18.88 (8.94)	19.19 (8.41)	18.84 (9.01)
6. Number of Inpatient Encounters in Last Six Months	0.62 (1.30)	1.17 (1.74)	0.55 (1.21)
7. Discharge Disposition			
Home/Self Care	45,931 (44.00)	3816 (31.25)	42,115 (45.68)
Home Care	23,290 (22.31)	3560 (29.15)	19,730 (21.40)
SNF	25,669 (24.59)	3668 (30.04)	22,001 (23.87)
Rehab	2948 (2.82)	367 (3.01)	2581 (2.80)
LTC, Federal Hospital	1783 (1.71)	463 (3.79)	1320 (1.43)
AMA	551 (0.53)	99 (0.81)	452 (0.49)
Others	4226 (4.05)	238 (1.95)	3988 (4.33)
8. Mean Albumin Level			
< 3.4 g/dl	54,177 (51.89)	8087 (66.23)	46,090 (50.00)
3.4–5.0 g/dl	20,535 (19.67)	1829 (14.98)	18,706 (20.29)
> 5	134 (0.13)	11 (0.09)	123 (0.13)
Unknown	29,552 (28.31)	2284 (18.70)	27,268 (29.58)
9. Leukemia Current	297 (0.28)	64 (0.52)	233 (0.25)
10. Leukemia History	1272 (1.22)	246 (2.01)	1026 (1.11)
11. Malignancy Current	5043 (4.83)	847 (6.94)	4196 (4.55)
12. Malignancy History	26,620 (25.50)	3924 (32.13)	22,696 (24.62)
13. RF without Hemo Current	14,061 (13.47)	2348 (19.23)	11,713 (12.71)
14. History of Alcohol Substance Abuse	20,641 (19.77)	2954 (24.19)	17,687 (19.19)
15. Dementia Current	3305 (3.17)	398 (3.26)	2907 (3.15)
16. Dementia History	15,559 (14.90)	2143 (17.55)	13,416 (14.55)
17. Trauma Current	7900 (7.57)	949 (7.77)	6951 (7.54)
18. Trauma History	50,428 (48.30)	6995 (57.28)	43,433 (47.11)

### Model training and evaluation

For both simulated and real data, 5 models were trained:

1. Autoencoder: logistic model applied to features generated by stacked sparse autoencoders
2. LASSO: logistic model with LASSO selection on raw predictors together with transformations of numeric predictors (if there are any)
3. Random Forest with raw predictors
4. Simple Reg: logistic model applied only to important variables
5. Enhanced Reg: the proposed strategy which applies logistic model to the new features in model 1 combined with important variables in model 4. We additionally use LASSO penalty to achieve a sparser model.

All analyses were performed with R 3.4.1 and SAS 9.4. We implemented autoencoder in H2O via R interface by using the R package ‘h2o’ [14]. To obtain sparse autoencoders, an L1 penalty was applied to the coefficients with respect to each hidden unit and the value of penalty parameter was chosen to be 10^− 4^. We decided to use two hidden layers for both simulated and real data. The number of hidden layers and number of units in each layer were determined by the overall predictive performance of models. For example, in the real EHR data, we tuned the number of new features generated by autoencoder with the value ranging from 50 to 300 and found that the predictive power of models increases with the number of features. The number of 200 was chosen because in this situation model performance was close to optimal while decently reducing the number of features. From results of simulation and real data application, autoencoder with two hidden layers has already achieved remarkable performance. Thus, we adopt the setting of two hidden layers throughout the numeric studies while to some extent reduce the possibility of overfitting raw data.

In LASSO, we set the L1 penalty parameter to the value at which the minimal cross-validated error was achieved (*λ*_*min*_) using the training data. As LASSO is designed for linear models, we report the results of LASSO after adding quadratic, cubic and log transformations of numeric predictors (if there are any) to both training and testing data. Note that no transformation is needed under scenarios 2, 3 and 4 of simulation study as there are only categorical variables. Simple Reg employs no additional feature selection. For random forest, we just adopt the default settings of function randomForest in R package ‘randomForest’, e.g., set number of trees to grow to the default value 500.

Our decision to use LASSO selection in Enhanced Reg is an attempt to remedy autoencoder’s unsupervised nature. Autoencoder captures variability in EHR data, which might or might not contribute to the response of Y. Therefore, we need another variable selection process for the final predictive model to get rid of redundant information, which can simplify the model and make it more robust.

During evaluation, we used precision given recall, positive predictive value (PPV) given negative predictive value (NPV) and AUC to measure the performance of predictive models. The definitions of these measures are all based on numbers of true/false positives and true/false negatives as listed in Table 2. We report precision given recall equal to 0.7. PPV is presented given NPV equal to 0.95 (simulated data and real data with the response variable Readmit30) or 0.99 (real data with the other four response variables that are rare events with high NPVs). AUC is an overall measure for the performance of predictive models for relatively common events. But note that it is not a good measure for rare events; instead, precision/recall or PPV/NPV can be a better choice. For all the above measures, higher is better, in general. In addition, we display the number of features in the model to measure the complexity of predictive models. A smaller number of features means the resulting model has a lower possibility to overfit raw data.

**Table 2:** Definition of true positive, false positive, true negative and false negative

		Predicted Value		Measures	
		1	0		
True Value	1	true positive (a)	false negative (c)	Sensitivity: a/(a + c)	Recall: a/(a + c)
	0	false positive (b)	true negative (d)	Specificity: d/(d + b)	
Measures		PPV: a/(a + b)	NPV: d/(c + d)		
		Precision: a/(a + b)			

## Results

### Simulation study results

Table 3 shows the performance of all methods under the four scenarios described in the simulation study design. Overall, predictive models using only new features generated from autoencoder are not the best but do have decent performance. By combining important variables with new features generated from autoencoder, Enhanced Reg achieves better results. In fact, Enhanced Reg is always the second-best performing strategy in scenarios 2–4, though LASSO exhibits the best overall predictive capability with the price of a much longer list of features. Under all scenarios, figures of Autoencoder and Random Forest are closely matched by the numbers of Enhanced Reg and LASSO, which is consistent with the finding in [29] that performances for well-established predictive models tend to be similar when sample size is large.

**Table 3:** Simulation study results. Mean and coefficient of variation (in parenthesis) of precision (when recall = 0.70), PPV (when NPV = 0.95), AUC, NO. (number of features in predictive models) of five prediction models in testing set in 100 repetitions

Scenarios	Prediction Models	Precision (%) (recall = 0.70)	PPV (%) (NPV = 0.95)	AUC	NO.
1. Raw Data	Autoencoder	24.23 (0.18)	19.93 (0.07)	0.749 (0.01)	50 (0.00)
	LASSO (*λ*_*min*_)	28.25 (0.17)	25.09 (0.05)	0.788 (0.01)	300 (0.06)
	Random Forest	25.63 (0.18)	21.93 (0.06)	0.767 (0.01)	100 (0.00)
	Simple Reg	20.96 (0.20)	15.73 (0.11)	0.708 (0.02)	12 (0.00)
	Enhanced Reg	24.62 (0.18)	20.45 (0.07)	0.754 (0.01)	57 (0.03)
2. Correct Categories	Autoencoder	25.07 (0.18)	21.45 (0.07)	0.757 (0.03)	50 (0.00)
	LASSO (*λ*_*min*_)	26.25 (0.17)	22.94 (0.05)	0.771 (0.01)	132 (0.02)
	Random Forest	24.93 (0.18)	21.57 (0.06)	0.759 (0.01)	136 (0.00)
	Simple Reg	21.36 (0.18)	17.10 (0.09)	0.713 (0.01)	16 (0.00)
	Enhanced Reg	25.77 (0.17)	22.32 (0.06)	0.766 (0.01)	60 (0.06)
3. Incorrect Categories	Autoencoder	22.73 (0.18)	18.82 (0.08)	0.732 (0.01)	60 (0.00)
	LASSO (*λ*_*min*_)	24.07 (0.17)	20.25 (0.06)	0.748 (0.01)	132 (0.02)
	Random Forest	22.70 (0.18)	18.67 (0.07)	0.733 (0.01)	136 (0.00)
	Simple Reg	19.83 (0.19)	15.31 (0.12)	0.690 (0.02)	16 (0.00)
	Enhanced Reg	23.61 (0.18)	19.69 (0.07)	0.743 (0.01)	69 (0.03)
4. Incorrect Categories and Missing Data	Autoencoder	24.16 (0.18)	20.45 (0.07)	0.748 (0.03)	60 (0.00)
	LASSO (*λ*_*min*_)	25.32 (0.17)	21.67 (0.06)	0.761 (0.01)	175 (0.08)
	Random Forest	23.61 (0.18)	19.92 (0.07)	0.745 (0.01)	226 (0.00)
	Simple Reg	20.92 (0.19)	16.31 (0.10)	0.706 (0.02)	28 (0.00)
	Enhanced Reg	24.89 (0.17)	21.25 (0.07)	0.756 (0.02)	81 (0.04)

Precision, PPV and AUC of Enhanced Reg remain roughly unchanged in the existence of categorization and missing data (scenario 2–4), and stand at 24.89, 21.25%, 0.756 in scenario 4, respectively. For results of Enhanced Reg, the biggest difference is observed between scenario 2 and scenario 3, where the above three measures decline by 2.16, 2.63, 2.30% due to incorrect categorization. Likewise, for the other four models, the numbers across all scenarios are quite stable, although the figures of LASSO drop from 28.25, 25.09%, 0.788 in scenario 1 to 24.07, 20.25%, 0.748 in scenario 3, correspondingly. LASSO tends to include more features in the final model than Enhanced Reg. In scenario 1, LASSO has number of features equal to 300 in contrast to 57 for Enhanced Reg, where predictive performance of the former beats the latter by a neck (28.25%, 0.788 in comparison with 24.62%, 0.754 for precision and AUC, respectively). In the most realistic setting, scenario 4, the number of features for LASSO are 94 greater than for Enhanced Reg (175 vs. 81) with a gain in evaluation measures no more than 0.5%.

By combining important variables with new features generated from autoencoder, Enhanced Reg achieves consistently better performance than using new features alone across all scenarios. Compared with Autoencoder, when all predictors were recoded to correct categories in scenario 2, Enhanced Reg sees an increase in the three measures of 0.70, 0.87 and 0.90% correspondingly. In scenario 4, by substituting Autoencoder with Enhanced Reg, the growth in precision, PPV and AUC is 0.73, 0.80, 0.80%, respectively.

### Real data results

Table 4 shows the results of the real EHR data analysis. Note that we used the same 469 predictors to build predictive models for five different response variables. Thus, during each repetition, the same 200 new features generated by autoencoder are applied to Autoencoder and Enhanced Reg for all responses. Across all five models, the measures of model performance for relatively rarer events, COPD, AMI, HF and Pneumonia, exceed those for Readmit30.

**Table 4:** Real data results. Mean and coefficient of variation (in parenthesis) of precision (when recall = 0.7), PPV (when NPV = 0.95 for Readmit 30 and 0.99 for the others), AUC, NO. (number of features in predictive models) of five prediction models in testing set in 100 repetitions

Response	Prediction Models	Precision (%) (recall = 0.7)	PPV (%) (NPV = 0.95/0.99)	AUC	NO.
Readmit30	Autoencoder	19.04 (0.02)	16.88 (0.02)	0.707 (0.01)	200 (0.00)
	LASSO (*λ*_*min*_)	19.70 (0.02)	17.79 (0.02)	0.719 (0.00)	162 (0.10)
	Random Forest	18.48 (0.02)	16.50 (0.02)	0.707 (0.01)	469 (0.00)
	Simple Reg	18.70 (0.02)	16.06 (0.02)	0.700 (0.01)	25 (0.00)
	Enhanced Reg	19.69 (0.02)	17.68 (0.02)	0.717 (0.00)	144 (0.10)
COPD	Autoencoder	55.90 (0.02)	42.16 (0.03)	0.961 (0.00)	200 (0.00)
	LASSO (*λ*_*min*_)	58.02 (0.02)	44.50 (0.02)	0.963 (0.00)	266 (0.04)
	Random Forest	56.19 (0.02)	40.45 (0.03)	0.956 (0.00)	469 (0.00)
	Simple Reg	51.51 (0.02)	35.53 (0.03)	0.952 (0.00)	21 (0.00)
	Enhanced Reg	57.06 (0.02)	43.62 (0.02)	0.962 (0.00)	161 (0.08)
AMI	Autoencoder	57.40 (0.04)	68.80 (0.03)	0.985 (0.00)	200 (0.00)
	LASSO (*λ*_*min*_)	58.57 (0.04)	70.10 (0.04)	0.986 (0.00)	64 (0.59)
	Random Forest	56.32 (0.03)	65.90 (0.03)	0.982 (0.00)	469 (0.00)
	Simple Reg	52.24 (0.04)	56.43 (0.06)	0.984 (0.00)	11 (0.00)
	Enhanced Reg	59.26 (0.04)	70.66 (0.03)	0.986 (0.00)	129 (0.14)
Heart Failure	Autoencoder	61.48 (0.02)	43.94 (0.02)	0.961 (0.00)	200 (0.00)
	LASSO (*λ*_*min*_)	63.15 (0.02)	45.88 (0.02)	0.964 (0.00)	195 (0.08)
	Random Forest	60.67 (0.02)	42.56 (0.02)	0.958 (0.00)	469 (0.00)
	Simple Reg	57.81 (0.02)	38.50 (0.02)	0.954 (0.00)	18 (0.00)
	Enhanced Reg	62.37 (0.02)	45.09 (0.02)	0.962 (0.00)	158 (0.10)
Pneumonia	Autoencoder	40.17 (0.03)	34.56 (0.03)	0.955 (0.00)	200 (0.00)
	LASSO (*λ*_*min*_)	42.18 (0.03)	35.94 (0.02)	0.958 (0.00)	204 (0.09)
	Random Forest	38.27 (0.03)	32.44 (0.03)	0.951 (0.00)	469 (0.00)
	Simple Reg	32.44 (0.02)	28.76 (0.02)	0.942 (0.00)	11 (0.00)
	Enhanced Reg	41.39 (0.03)	35.54 (0.02)	0.957 (0.00)	173 (0.08)

Enhanced Reg is the best-performing model when response is AMI, or otherwise the second-best strategy with performance slightly worse than LASSO. With response variable Readmit30, COPD, HF and Pneumonia, the average number of features for LASSO are greater than Enhanced Reg. By contrast, with the response variable AMI, the number of features for Enhanced Reg double the amount of LASSO. Nevertheless, it is worth mentioning that, in this case, the CV (coefficient of variation) of number of features for LASSO is 0.59, in marked contrast to 0.14 for Enhanced Reg, which may indicate a lack of robustness in LASSO models.

Applying logistic model only to new features generated by autoencoder gives decent performance and incorporating response-specific variables (Enhanced Reg) further enhances performance. When response is readmit30, Enhanced Reg increases the AUC from 0.707 (Autoencoder) to 0.717 (Enhanced Reg). At the same time, the number of features of the model are reduced from 200 to 144 due to the shrinkage effect of LASSO selection. For other response variables, we observe minimum changes to AUC, but AUC for all methods is already greater than 0.942 due to the low occurrence of positive events. Enhanced Reg also leads to an increment in precision of 0.66% (for Readmit30), 1.16% (for COPD), 1.86% (for AMI), 0.89% (for HF) or 1.22% (for pneumonia).

## Discussion

A potential usage of the new features generated by autoencoder is to create a set of standardized variables that represent most of the variations in EHR data. These standardized variables are capable of being widely used in a variety of predictive models. Another way to utilize the new representation is to define distances between patients/encounters so that a comparable control group can be easily extracted from the data [31, 32].

Since representations are not limited to specific usage, to some degree, new features from autoencoder may have a lower chance to overfit data even without bootstrap-based or other cross validation approaches when modeling. According to [33], a simple, robust model should be preferred to an overly fine-tuned model for the specific data.

From another point of view, these new features represent the overall variation of predictors but potentially fail to capture the information most relevant to the response. Therefore, we came up with the idea of incorporating some response-specific important variables to aid with predictive modeling. Important variables, usually originating from expert experience or research, contain useful response-specific information. Using both the response-specific information and general representations of all predictors from autoencoder, we are likely to derive accurate and generalizable predictive models. In simulation studies, Enhanced Reg shows decent performance with a much shorter list of features compared to LASSO, which inspired us to apply this strategy to real data analysis. The results in real data further support the validity of this approach. However, it is tricky to define how many important variables are ‘enough’ for the purpose of enhancing predictive performance. In addition, it is worth researching other strategies for combining the response-specific information.

In real applications, we are always facing the tradeoff between the ease of use and the accuracy of prediction. New features from autoencoder only represent generic variation among predictors, enabling wide applicability to various modeling tasks and potentially mitigating the labor of extracting specialized datasets. Still, features generated by unsupervised strategies may or may not capture the information most related to the variation of specific response variables. From our simulation and real data analysis, the predictive performance of Enhanced Reg is to some extent inferior to LASSO. Regardless of the nature of unsupervised features, it may also be partially due to the incompleteness of our dataset since we only extracted a small number of variables for each patient. Consequently, features from autoencoder may not draw a whole picture of each subject. In contrast, as a supervised strategy, LASSO identifies the predictors that are most related to the response while penalizing the coefficients of less relevant predictors to zero. During modelling, we choose the value of penalty parameter via 10-fold cross validation. That is, the comparison is essentially between the ‘best’ model that LASSO could achieve with Enhanced Reg. In this circumstance, the proposed strategy tends to obtain a more parsimonious model under the limited scenarios of studies of this paper. Nevertheless, more experiments are still required to verify that this tendency persists in external data.

Another concern about features from autoencoder lies in its interpretability. Autoencoder exerts a series of nonlinear transformations on raw predictors to derive representations, resulting in new features’ vague interpretation of original variables. On the other hand, vague interpretation of features extracted from autoencoder could have an upside. Since these features do not directly represent traditional patient characteristics or identifying features, they can obscure protected health information (PHI) and may provide an ethical alternative for sharing data across external institutions and research studies. Increased sharing would enable repeatable results and broader exploration, consequently improving quality and accountability in clinical research.

## Conclusions

In this paper, we have explored the potential usage of autoencoder features extracted from EHR data in prediction models. Autoencoder features alone in logistic models have decent, though not optimal, prediction performance in our examples. To enhance the performance, we proposed a strategy, Enhanced Reg, that combines generic features generated from autoencoder with response-specific predictors with established predictive importance. Enhanced Regression achieves better performance than the strategy of using autoencoder features alone. In simulation studies, Enhanced Reg has decent performance though LASSO exhibits the best overall predictive performance with the price of much larger number of features in the final model. The results in simulation and real data analysis indicate the possibility of applying standardized features from autoencoder and the enhanced regression strategy across a wide range of responses, with potential gains in efficiency, portability, and responsible data sharing.

## Supplementary information

**Additional file 1: Figure S1.** Examples of the multistage functions used in simulation studies. **Figure S2.** Examples of the continuous functions used in simulation studies in Appendix.

## Data Availability

The datasets used and/or analyzed during the current study are available from the corresponding author on reasonable request.

## Abbreviations

AMI: Acute myocardial infarction
AUC: Area under the receiver operating characteristic curve
COPD: Chronic obstructive pulmonary disease
EHR: Electronic health record
HF: Heart failure
LASSO: Least absolute shrinkage and selection operator
NPV: Negative predictive value
PPV: Positive predictive value
Readmit30: 30-day readmission rate
