# Supplementary material for: The application of unsupervised deep learning in predictive models using electronic health records
Source: BMC Med Res Methodol. 2020 Feb 26;20:37. doi: 10.1186/s12874-020-00923-1 (PMC7043035; doi:10.1186/s12874-020-00923-1)
Supplement: Supplementary file 1 — Additional file 1: Figure S1. Examples of the multistage functions used in simulation studies. Figure S2. Examples of the continuous functions used in simulation studies in Appendix. [file 12874_2020_923_MOESM1_ESM.docx]

**Appendix**

Figure 1: examples of the multistage functions used in simulation studies.


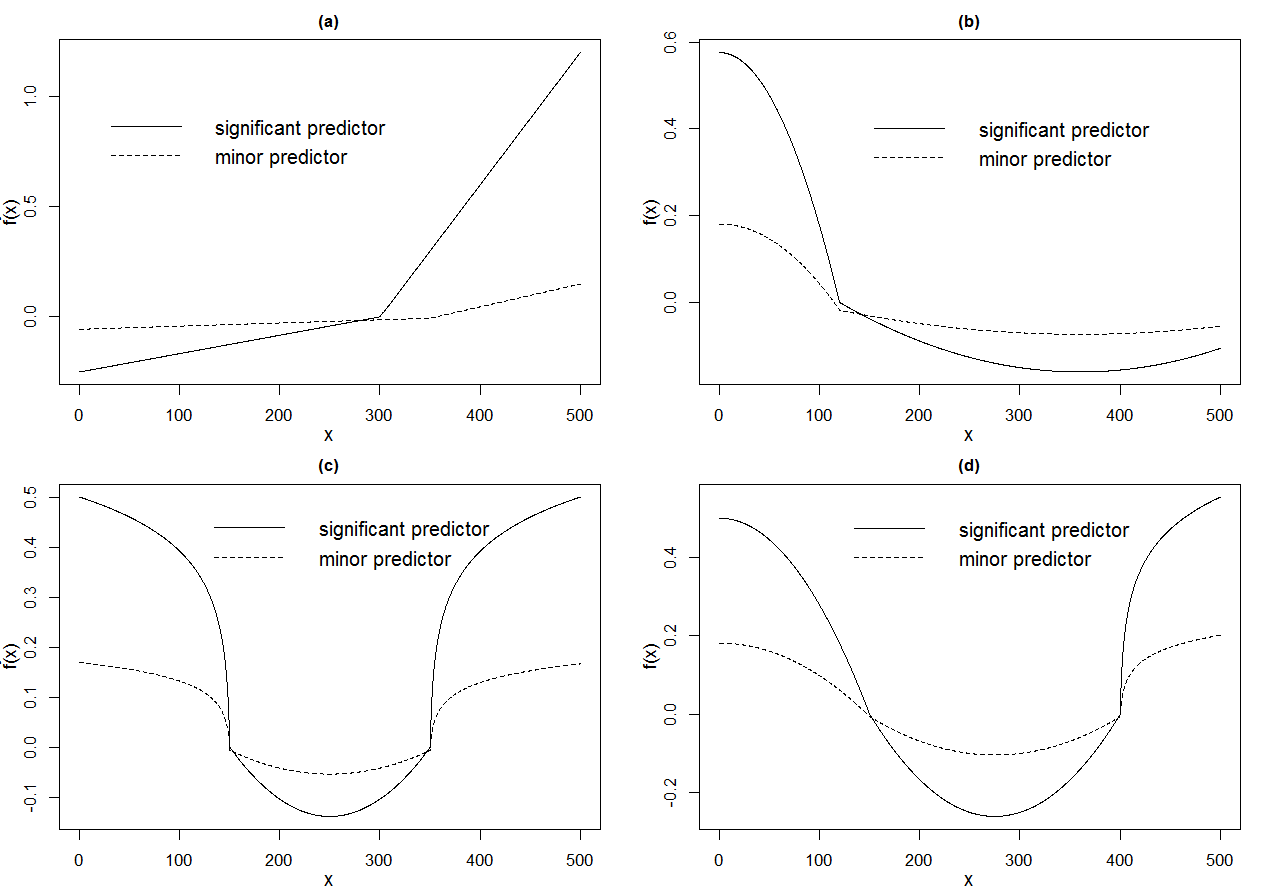


For (a) in Figure 1, the multistage functions for significant predictors are:

$$f\left( x \right)=\left\{ \begin{aligned} \frac{1}{1200}\left( x-300 \right),x\leq300 \\ \frac{3}{500}\left( x-300 \right),x>300 \end{aligned} \right.$$

For (a) in Figure 1, the multistage functions for minor predictors are:

$$f\left( x \right)=\left\{ \begin{aligned} \frac{1.725}{12000}\left( x-350 \right)-0.006,x\leq350 \\ \frac{10.35}{10000}\left( x-350 \right)-0.006,x>350 \end{aligned} \right.$$

For (b) in Figure 1, the multistage functions for significant predictors are:

$$f\left( x \right)=\left\{ \begin{aligned} \frac{1}{360000}(x-120)\left( x-600 \right),x\geq120 \\ \frac{1}{25000}\left( {120}^{2}-x^{2} \right),x<120 \end{aligned} \right.$$

For (b) in Figure 1, the multistage functions for minor predictors are:

$$f\left( x \right)=\left\{ \begin{aligned} \frac{1.15}{1200000}\left( x-120 \right)\left( x-600 \right)-0.018,x\geq120 \\ \frac{3.45}{250000}\left( {120}^{2}-x^{2} \right)-0.018,x<120 \end{aligned} \right.$$

For (c) in Figure 1, the multistage functions for significant predictors are:

$$f\left( x \right)=\left\{ \begin{aligned} \frac{1}{10}\log\left( 151-x \right),x<150 \\ \frac{1}{72000}\left( x-150 \right)\left( x-350 \right),150\leq x\leq350 \\ \frac{1}{10}\log\left( x-349 \right),x>350 \end{aligned} \right.$$

For (c) in Figure 1, the multistage functions for minor predictors are:

$$f\left( x \right)=\left\{ \begin{aligned} \frac{3.45}{100}\log\left( 151-x \right)-0.003,x<150 \\ \frac{2.3}{480000}\left( x-150 \right)\left( x-350 \right)-0.006,150\leq x\leq350 \\ \frac{6.9}{200}\log\left( x-349 \right)-0.006,x>350 \end{aligned} \right.$$

For (d) in Figure 1, the multistage functions for significant predictors are:

$$f\left( x \right)=\left\{ \begin{aligned} \frac{1}{45000}\left( {150}^{2}-x^{2} \right),x<150 \\ \frac{1}{60000}\left( x-150 \right)\left( x-400 \right),150\leq x\leq400 \\ \frac{3}{25}\log\left( x-399 \right),x>400 \end{aligned} \right.$$

For (d) in Figure 1, the multistage functions for minor predictors are:

$$f\left( x \right)=\left\{ \begin{aligned} \frac{1}{120000}\left( {150}^{2}-x^{2} \right)-0.006,x<150 \\ \frac{0.75}{120000}\left( x-150 \right)\left( x-400 \right)-0.006,150\leq x\leq400 \\ \frac{4.5}{100}\log\left( x-399 \right)-0.006,x>400 \end{aligned} \right.$$

Figure 2: examples of the continuous functions used in simulation studies.


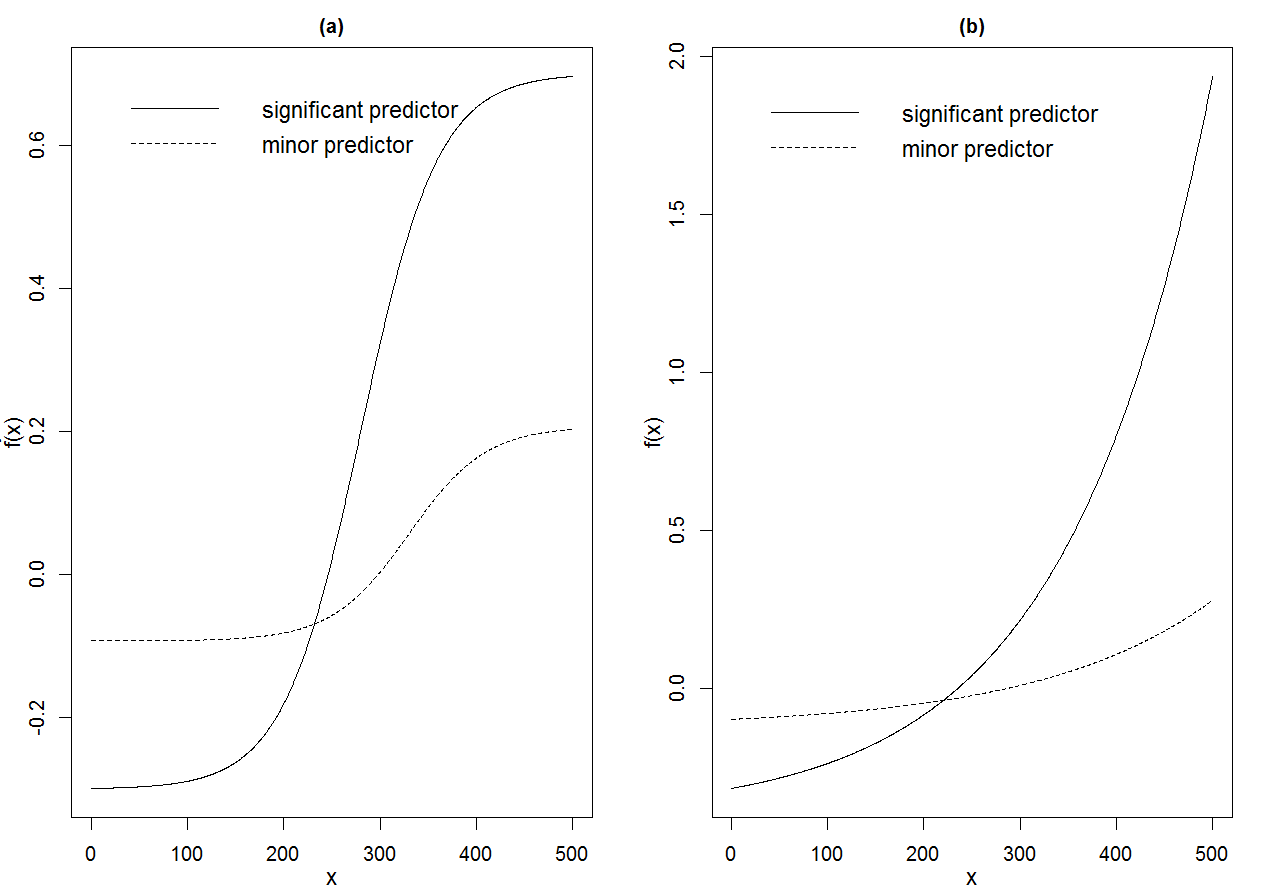


For (a) in Figure 2, the continuous functions are:

$$f\left( x \right)=\left\{ \begin{aligned} \frac{exp\left( \frac{x}{40}-7 \right)}{\left( 1+exp\left( \frac{x}{40}-7 \right) \right)}-0.3,for significant predictors \\ \frac{0.3exp\left( \frac{x}{40}-8.25 \right)}{\left( 1+exp\left( \frac{x}{40}-8.25 \right) \right)}-0.093,for minor predictors \end{aligned} \right.$$

For (b) in Figure 2, the continuous functions are:

$$f\left( x \right)=\left\{ \begin{aligned} \frac{1}{12}exp\left( \frac{x}{150} \right)-0.4,for significant predictors \\ \frac{1}{40}exp\left( \frac{x}{180} \right)-0.123,for minor predictors \end{aligned} \right.$$
